# Syndecan-1 mediates internalization of apoE-VLDL through a low density lipoprotein receptor-related protein (LRP)-independent, non-clathrin-mediated pathway

**DOI:** 10.1186/1476-511X-5-23

**Published:** 2006-08-31

**Authors:** Larissa C Wilsie, Amanda M Gonzales, Robert A Orlando

**Affiliations:** 1 Department of Biochemistry and Molecular Biology, University of New Mexico, School of Medicine, MSC08 4670 1 University of New Mexico, Albuquerque, New Mexico, 87131, USA

## Abstract

**Background:**

Triacylglyerol-rich very low density lipoprotein (VLDL) particles are the primary carriers of fatty acids in the circulation and as such serve as a rich energy source for peripheral tissues. Receptor-mediated uptake of these particles is dependent upon prior association with apolipoprotein E (apoE-VLDL) and is brought about by cell surface heparan sulfate proteoglycans (HSPG) in some cell types and by the low density lipoprotein receptor-related protein (LRP) in others. Although LRP's role in apoE-VLDL uptake has been well studied, the identity of the HSPG family member that mediates apoE-VLDL uptake has not been established. We investigated if syndecan-1 (Syn-1), a transmembrane cell surface HSPG, is able to mediate the internalization of apoE-VLDL and examined the relationship between Syn-1 and LRP toward apoE-VLDL uptake. For this study, we used a human fibroblast cell line (GM00701) that expresses large amounts of LRP, but possesses no LDL receptor activity to eliminate its contributions toward apoE-VLDL uptake.

**Results:**

Although LRP in these cells is fully active as established by substantial α_2_macroglobulin binding and internalization, uptake of apoE-VLDL is absent. Expression of human Syn-1 cDNA restored apoE-VLDL binding and uptake by these cells. Competition for this uptake with an LRP ligand-binding antagonist had little or no effect, whereas co-incubation with heparin abolished apoE-VLDL internalization. Depleting Syn-1 expressing cells of K^+^, to block clathrin-mediated endocytosis, showed no inhibition of Syn-1 internalization of apoE-VLDL. By contrast, treatment of cells with nystatin to inhibit lipid raft function, prevented the uptake of apoE-VLDL by Syn-1.

**Conclusion:**

These data demonstrate that Syn-1 is able to mediate apoE-VLDL uptake in human fibroblasts with little or no contribution from LRP and that the endocytic path taken by Syn-1 is clathrin-independent and relies upon lipid raft function. These data are consistent with previous studies demonstrating Syn-1 association with lipid raft domains.

## Background

Fatty acids, triacylglycerols, and cholesterol in plasma originate primarily from two sources; dietary intake and that which is synthesized and secreted by hepatocytes. Dietary lipids circulate in the form of chylomicrons which are synthesized by intestinal epithelial cells. They are relatively short lived particles during the postprandial period as they are rapidly metabolized to remnant lipoproteins and cleared from the circulation primarily by hepatocytes. By contrast, the liver synthesizes triacylglycerol-rich lipoproteins in the form of very low density lipoprotein (VLDL) particles which are much longer-lived fatty acid carriers than remnant lipoproteins. Because of this, VLDL particles provide the largest single source of fatty acids for peripheral tissues found in the circulation. These fatty acids are a rich energy source for cells with high metabolic rates and are also stored by adipose tissue for mobilization during periods of fasting. Previous studies have also shown that VLDL is highly atherogenic since excessive uptake of these lipoproteins by macrophages causes massive cholesterol accumulation and foam cell formation [1-3]. Moreover, elevated levels of VLDL are found in the plasma of patients with type III hyperlipoproteinemia [4]. It follows from these observations that cardiovascular health requires controlled levels of VLDL particles in the circulation, whether by decreased synthesis or increased uptake.

Considerable evidence has been presented suggesting that the mechanism for VLDL particle clearance involves cell surface binding and endocytic activities of either heparan sulfate proteoglycans (HSPG) [5-7] or the low density lipoprotein receptor-related protein (LRP) [8,9], or both receptors acting in a synergistic manner at the cell surface [10]. Receptor-mediated association of VLDL with the cell surface also requires enrichment of the particle with apolipoprotein E (apoE) [6,11]. Evidence supporting a role for LRP in apoE-VLDL uptake has been gathered by blocking LRP's endocytic function with a ligand binding antagonist or by tissue-specific gene ablation, both of which result in increased circulating levels of VLDL particles [12,13]. HSPG-mediated internalization of apoE-VLDL has been reported in several independent cell culture systems including fibroblasts [14-16], CHO cells [17], HepG2 cells [18,19], macrophages [20], and vascular smooth muscle cells [21]. In each of these cell types, a significant reduction in apoE-VLDL internalization was demonstrated following the inhibition of HSPG activity either through a coincubation with heparin or heparinase treatment prior to ligand binding. Moreover, intravenous administration of heparinase into the portal circulation reduced hepatocyte-mediated VLDL uptake by 70% [7,10]. Notably, in contrast to the studies examining LRP's role in apoE-VLDL clearance, studies on HSPG report that a coincubation of labeled apoE-VLDL with an LRP ligand binding antagonist showed minimal effects on lipoprotein uptake indicating little or no participation by this receptor. These results have created controversy as to the identity of the receptor used by VLDL particles for endocytic clearance. To distinguish which path, HSPG or LRP, serves as the primary uptake mechanism for apoE-VLDL, we have recently re-examined apoE-VLDL endocytosis following the inhibition of HSPG maturation using reagents that prevent glycosaminoglycan chain addition [16]. In this study, we showed that apoE-VLDL uptake occurs primarily through an HSPG-mediated process with little or no contributions from LRP.

Identifying the HSPG that mediates apoE-VLDL clearance has been elusive. Specific HSPG, like those of the syndecan [22,23], glypican [23,24] and perlecan [25,26] families, have primarily been studied in the context of their abilities to modulate growth factor responses. Although, syndecan [27,28] and perlecan [29,30] have been shown to internalize modified LDL, little information is available identifying the HSPG responsible for apoE-VLDL uptake. Syndecan-1 (Syn-1) was recently shown to colocalize with chylomicron remnants at the surface of hepatocytes suggesting that it may participate in the endocytic clearance of dietary lipids [31]. In the present study, we investigated if Syn-1 is capable of mediating apoE-VLDL uptake and examined its functional relationship with LRP in lipoprotein handling at the cell surface.

## Results

**Figure 1:**
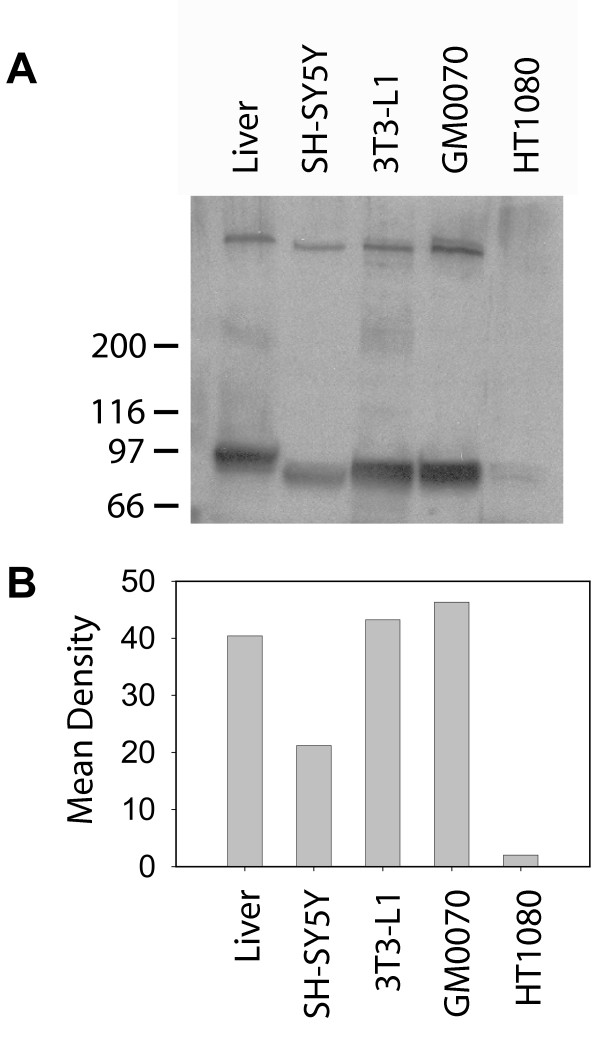
**Comparison of LRP expression in various cell lines**. (A) Cell lysates were prepared using 1% Triton X-100 detergent, proteins (20 μg/lane) were separated by 6% SDS-PAGE, and immunoblotted with anti-LRP pAb raised against a synthetic peptide derived from the cytoplasmic tail sequence [59]. Bound antibodies were detected by chemiluminescence imaging. Rat liver extract was used as a positive control for LRP expression. (B) Densitometric scans of the 95 kD light chain of LRP in (A).

To determine if Syn-1 is capable of endocytic uptake of apoE-VLDL and to examine the relationship between Syn-1 and LRP toward apoE-VLDL clearance, we searched for a cell line that 1) expresses no detectable Syn-1 to permit transfection studies with human Syn-1, 2) expresses high levels of LRP to permit accurate and reliable measurements of its endocytic properties, and 3) expresses no LDL receptor activity to remove its contribution toward apoE-VLDL binding and clearance that might complicate our measurements of Syn-1 and LRP activities. Our search led us to the GM00701 human cell line which was obtained from a patient diagnosed with familial hypercholesterolemia and expresses LDL receptors with < 1% of normal activity. We compared LRP expression in this cell line to other cell lines known to express this receptor, including human SH-SY5Y neuroblastoma [32], mouse pre-adipocyte 3T3-L1 [33] and human HT1080 fibrosarcoma [34]. By semi-quantitative immunoblot analysis, we found that the GM00701 cells express more LRP than other cultured cells previously used to study LRP's cellular activities (Fig. 1). Interestingly, the relative molecular mass of LRP's light chain (~95 kDa) is slightly smaller in the cultured cell lines as compared to liver tissue, which is the *in vivo *location for the highest level of LRP expression. This is likely due to post-translational processing since no alternatively spliced transcripts for LRP have been reported.

**Figure 2:**
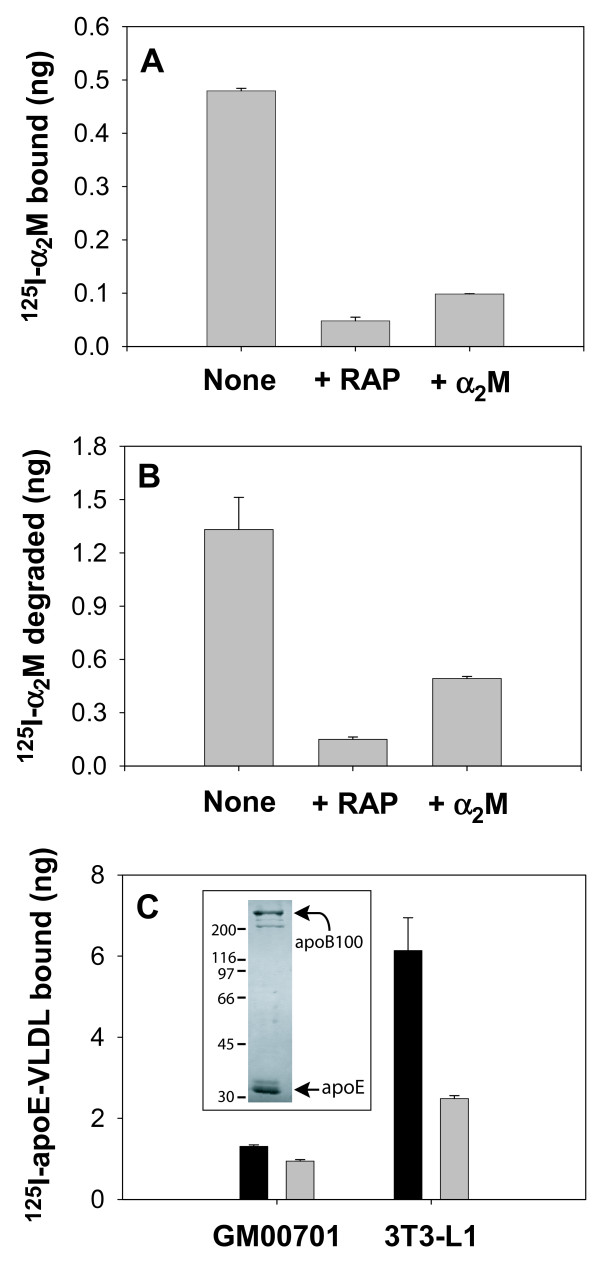
**^125^I-α_2_M and ^125^I-apoE-VLDL handling by GM00701 cells**. GM00701 cells were incubated with ^125^I-α_2_M (1 μg/ml) at 4°C for 3 h (A) or at 37°C for 1 h (B) in the absence or presence of recombinant human RAP-GST (50 μg/ml) or unlabeled α_2_M (10 μg/ml). (C) GM00701 and 3T3-L1 cells were incubated with ^125^I-apoE-VLDL (2 μg/ml) supplemented with 3 μg/ml apoE at 4°C for 3 h in the absence (solid bars) or presence (shaded bars) of unlabeled apoE-VLDL (100 μg/ml). After the indicated incubation time, ^125^I-ligand bound to cells at 4°C was quantitated by scintillation counting following extraction of cells with 0.1 M NaOH. ^125^I-α_2_M degradation was quantitated following TCA precipitation as described in Methods. Error bars represent standard deviations of triplicate points. Inset, purified VLDL was separated by 7.5% SDS-PAGE and proteins were stained with Coomassie R. Molecular mass markers shown at left are in kD.

Since GM00701 cells express large amounts of LRP, we anticipated that they would have a substantial capacity to bind and degrade the LRP specific ligand, α_2_-macroglobulin (α_2_M). To test this assumption, GM00701 cells were incubated at 4°C with ^125^I-α_2_M in the absence or presence of unlabeled α_2_M or the LRP ligand binding antagonist, receptor associated protein (RAP). A significant amount of α_2_M bound to GM00701 cells (Fig. 2A), of which 80–90% was specific as determined by competition with unlabeled α_2_M and RAP. When the same experiment was performed at 37°C to measure LRP-specific ligand internalization and degradation, we found that GM00701 cells degraded more ^125^I-α_2_M than the binding capacity at the cell surface suggesting not only efficient LRP internalization, but also rapid recycling (Fig. 2B).

Since LRP has previously been reported to bind apoE-VLDL particles [8,9], we investigated if GM00701 cells were able to efficiently bind ^125^I-apoE-VLDL with its high expression level of LRP. To accomplish this, we isolated VLDL particles from plasma obtained from New Zealand White rabbits maintained on a high fat, high cholesterol diet. Prior to radiolabeling, the VLDL preparation was analyzed by SDS-PAGE followed by Coomassie staining to confirm that these particles contain the expected apolipoproteins, B100 and E. As shown in Figure 2C (inset), the purified VLDL particles do indeed contain apoB100 (Mr, 512 kD) and apoE (Mr, 34 kD) indicating that they have the proper associated proteins necessary for efficient receptor binding and internalization. When GM00701 cells were incubated at 4°C with ^125^I-apoE-VLDL, we found little or no specific binding (Fig. 2C) indicating that these cells are incapable of binding apoE-VLDL in spite of the presence of endocytically active LRP. Cell surface binding activity of our ^125^I-apoE-VLDL preparation was confirmed by high level specific binding to mouse pre-adipocyte 3T3-L1 cells [35].

**Figure 3:**
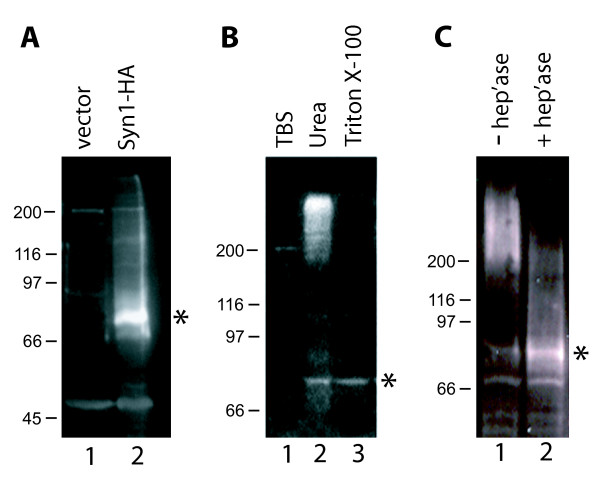
**Expression of syndecan-1 in GM00701 cells**. (A) GM00701 cells were transfected with pMH alone (lane 1) or pMH/Syn-1-HA (lane 2). Forty eight hours after transfection, total protein was extracted from cells with 1% Triton X-100 and equal amounts of protein (20 μg/lane) were separated by 7.5% SDS-PAGE, transferred to PVDF membrane and immunoblotted with anti-Syn-1 polyclonal antisera (1:1000) that was raised against a recombinant his-tagged fusion protein representing full length human Syn-1. Chemiluminescence detection was used to visualize bound antibodies. (B) GM00701 cells were transfected with pMH/Syn-1-HA and cells were selected that stably express Syn-1 (GM00701/Syn-1-HA). Cells were incubated with either TBS (lane 1), 8 M urea (lane 2), or 1% Triton X-100 (lane 3). Insoluble material was pelleted by centrifugation and soluble proteins were separated by 7.5% SDS-PAGE and immunoblotted with anti-Syn-1 antisera as described in (A). (C) GM00701/Syn-1-HA cells were incubated without (lane 1) or with (lane 2) heparinase (1 unit/ml) for 5 h at 37°C. Total cellular proteins were extracted with 8 M urea, separated by 6% SDS-PAGE and immunoblotted with anti-Syn-1 antisera as described in (A). Asterisks indicate the 77 kD, non-glycosylated Syn-1 core protein [36, 37].

The inability of GM00701 fibroblasts to bind ^125^I-apoE-VLDL and the lack of Syn-1 expression as determined by immunoblot and RT-PCR analysis (data not shown), established these cells as a viable model for us to investigate the functional relationship between Syn-1 and LRP toward apoE-VLDL binding and clearance. Toward this end, we constructed a mammalian expression vector containing the full length human Syn-1 cDNA (pMH/Syn-1-HA) and carried out transient transfection studies with GM00701 cells. Immunoblot analysis of cells transfected with vector alone (Fig. 3A, lane 1) showed no endogenous expression of Syn-1. By contrast, cells transfected with pMH/Syn-1-HA (lane 2) demonstrated efficient Syn-1 expression (Mr ~77kDa). The 77 kDa protein identified by immunoblotting represents the core protein of Syn-1 [36,37]. Mature Syn-1 demonstrates a relative molecular mass of > 250 kD as a result of its multiple glycosaminoglycan additions [38].

In order to confirm proper maturation of Syn-1 in transfected cells as defined by heparan sulfate glycosaminoglycan addition, we transfected GM00701 cells with pMH/Syn-1-HA vector and enriched for those cells stably expressing Syn-1 using antibiotic selection. We then immunoblotted protein extracts from GM00701/Syn-1-HA cells prepared with 8 M urea. Urea extraction is often necessary to release mature Syn-1 from cytoskeletal interactions that are resistant to mild detergents such as Triton X-100 [37,39]. As shown in Figure 3B, a high molecular mass protein (Mr, > 250 kD) was identified by anti-Syn-1 antibodies following urea extraction (lane 2), but not after extraction with Triton X-100 (lane 3). As expected, treatment with Tris-buffered saline was unable to extract Syn-1 from cells (lane 1) since Syn-1 is an integral membrane protein.

To verify that the large molecular mass change in Syn-1 as seen by immunoblotting is due to heparan sulfate glycosaminoglycan addition, we investigated the effect of heparinase treatment on the electrophoretic mobility of Syn-1 from GM00701/Syn-1-HA cells. Without heparinase treatment, mature Syn-1 migrated to its expected relative molecular mass of > 250 kDa (Fig. 3C, lane 1). With heparinase treatment, a significant increase in Syn-1 core protein (Mr, 77 kDa) was observed that directly corresponded to a decrease in mature Syn-1 (Mr, > 250 kDa) (lane 2). We interpret these data to indicate that mature Syn-1 expressed by GM00701/Syn-1-HA cells contains heparan sulfate glycosaminoglycan chains and undergoes normal post-translational modifications.

**Figure 4:**
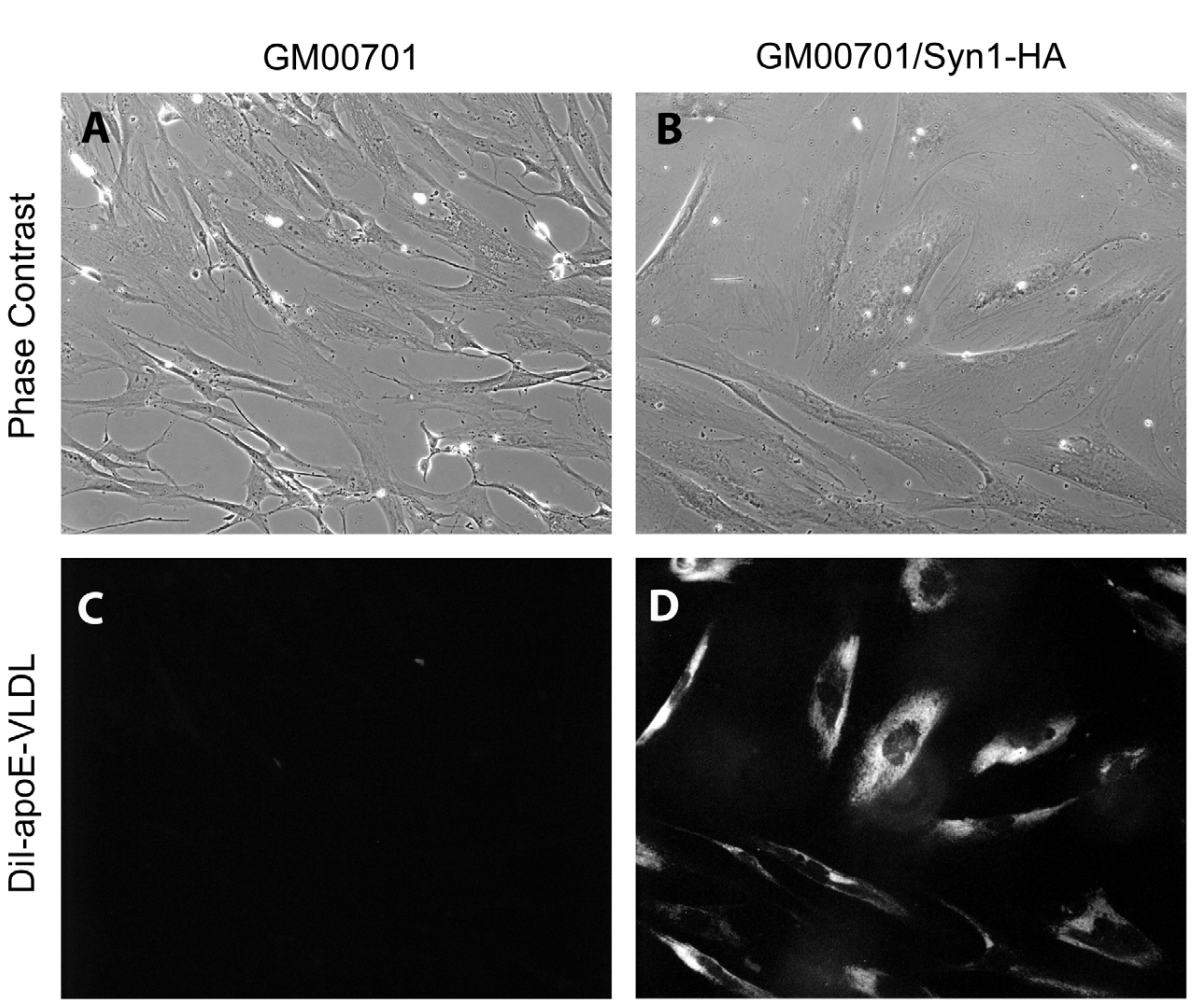
**Expression of Syn-1 restores apoE-VLDL uptake in GM00701 cells**. GM00701 (panels A and C) and GM00701/Syn-1-HA (panels B and D) cells were incubated with DiI-labeled apoE-VLDL (4 μg/ml) for 3 h at 37°C. Unassociated ligand was removed by rinsing, cells were fixed with paraformaldehyde and observed by fluorescence microscopy (panels C and D, 550 nm excitation-573 nm emission). Corresponding phase contrast images are shown in panels A and B. Magnification, 630X.

**Figure 5:**
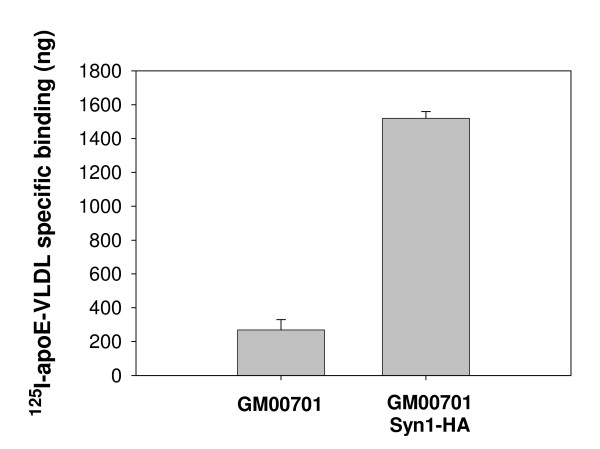
**Comparison of ^125^I-apoE-VLDL binding to GM00701 and GM00701/Syn-1-HA**. GM00701 or GM00701/Syn-HA cells were incubated with ^125^I-apoE-VLDL (2 μg/ml) for 3 h at 4°C in the absence or presence of unlabeled apoE-VLDL (100 μg/ml). Unbound ligand was removed by rinsing, cells were solubilized with 0.1 M NaOH and subjected to scintillation counting. Values shown represent specific ^125^I-apoE-VLDL binding and were obtained by subtracting amounts of ^125^I-apoE-VLDL bound in the presence of unlabeled apoE-VLDL from total ^125^I-apoE-VLDL binding. Error bars represent standard deviations of triplicate points.

Since GM00701 cells are unable to bind apoE-VLDL even with high expression levels of LRP, we next asked if introducing Syn-1 expression is able to rescue apoE-VLDL uptake by these cells. To address this, GM00701 and GM00701/Syn-1-HA cells were incubated with DiI-labeled apoE-VLDL at 37°C and examined by fluorescence microscopy for uptake of the labeled ligand. Consistent with the results we obtained in 4°C binding studies with ^125^I-apoE-VLDL, GM00701 cells were unable to bind or internalize DiI-apoE-VLDL (Fig. 4, panel C). However, GM00701/Syn-1-HA cells were able to readily take up DiI-apoE-VLDL as evidenced by labeled apoE-VLDL within intracellular vesicles (Fig. 4, panel D). These data clearly show that expression of Syn-1 rescues apoE-VLDL internalization by these cells. To measure the increase in apoE-VLDL binding resulting from Syn-1 expression, we incubated GM00701 and GM00701/Syn-1-HA cells with ^125^I-apoE-VLDL at 4°C in the absence or presence of unlabeled apoE-VLDL and quantitated specific ligand binding. Expression of Syn-1 in GM00701/Syn-1-HA cells resulted in a 5-fold increase in specific apoE-VLDL binding as compared to control GM00701 cells (Fig. 5).

**Figure 6:**
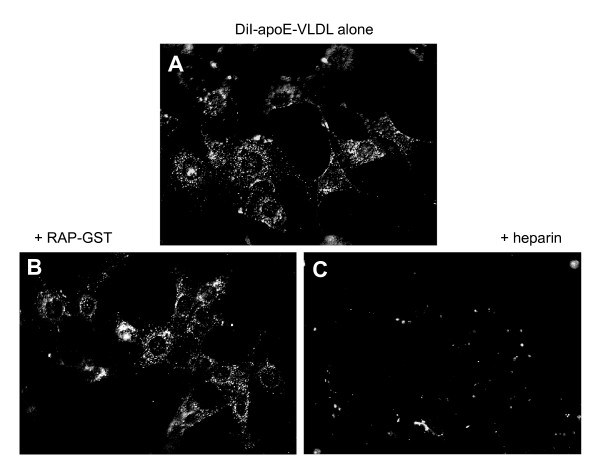
**Heparin, but not RAP-GST, competes for DiI-apoE-VLDL uptake by GM00701/Syn-1-HA cells**. GM00701/Syn-1-HA cells were cultured on glass coverslips and incubated with DiI-labeled apoE-VLDL (4 μg/ml) in the absence (panel A) or presence of recombinant human RAP-GST (50 μg/ml, panel B) or heparin (200 μg/ml, panel C) at 37°C for 3 h. Cells were fixed and processed for fluorescence microscopy. Magnification, 630X.

The question that arises from these observations asks if LRP plays a role with Syn-1 in the binding and uptake of apoE-VLDL. To address this question, we incubated GM00701/Syn-1-HA cells with DiI-apoE-VLDL in the absence or presence of either RAP-GST (to block LRP function) or heparin (to block Syn-1 function) and visualized ligand uptake by fluorescence microscopy following a 37°C incubation. RAP is known to prevent the binding of all ligands to LRP [40], including VLDL [41], and has proven invaluable as a pharmacological antagonist for characterizing LRP's ligand binding and uptake properties. With no co-incubation by ligand binding antagonists, DiI-apoE-VLDL is readily taken up by GM00701/Syn-1-HA cells as was originally identified in Fig. 4 (Fig. 6, panel A). Co-incubation with heparin blocked most, if not all, DiI-apoE-VLDL uptake (Fig. 6, panel C) demonstrating the requirement for heparan sulfate glycosaminoglycans on Syn-1 for DiI-apoE-VLDL uptake. However, RAP-GST demonstrated only marginal inhibition of uptake (Fig. 6, panel B), suggesting that Syn-1 is necessary for apoE-VLDL uptake and is able to internalize apoE-VLDL independent from LRP's endocytic activity.

**Figure 7:**
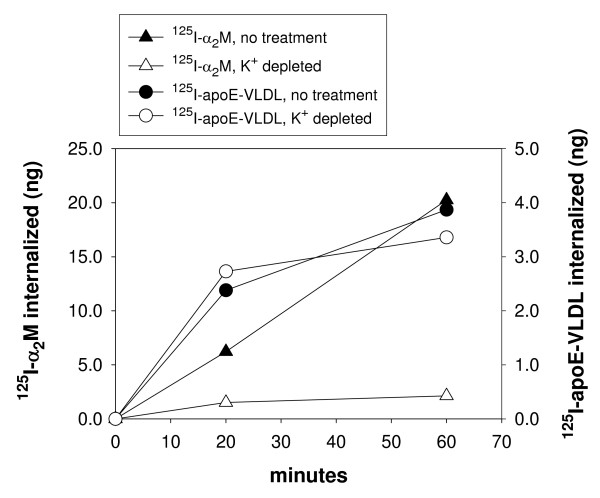
**^125^I-α_2_M internalization, but not ^125^I-apoE-VLDL, is inhibited by K^+ ^depletion of cells**. GM00701/Syn-1-HA cells were treated with K^+^-depleted buffers (open symbols), or K^+^-containing serum-free media (closed symbols), as described in Methods. Cells were incubated with either ^125^I-apoE-VLDL (2 μg/ml) (circles) or ^125^I-α_2_M (0.5 μg/ml) (triangles) for 20 min at 37°C. Specificity of uptake was determined by a parallel incubation with either unlabeled apoE-VLDL (100 μg/ml) or unlabeled α_2_M (5 μg/ml), respectively. After the 37°C incubation, cells were chilled to 4°C and acid stripped with 50 mM glycine, pH 3, 100 mM NaCl for 5 min to remove surface bound ligand (that which was not internalized). Internalized ligand was quantitated by solubilizing cells with 0.1 N NaOH and scintillation counting.

We [16] and others [14,18-21] have previously shown that internalization of apoE-VLDL by cells is partially mediated by HSPG and in the present study we show that Syn-1 is capable of serving the role of this HSPG. These data now raise important questions as to the endocytic pathway used for internalization. LRP, like other members of the LDL receptor family, contains the clathrin-mediated internalization signal, NPXY amino acid sequence in their cytoplasmic tails. However, transmembrane HSPG such as Syn-1 do not contain this sequence and are likely internalized by cells through a non-clathrin mediated pathway. Previous studies have shown that antibody induced clustering of cell surface Syn-1 promotes its association with detergent-insoluble lipid rafts [28]. In addition, cholesterol depletion of cells prevented Syn-1 from associating with these lipid rafts. Together, these observations suggest a role for non-clathrin-mediated pathways for Syn-1 internalization. To examine the pathway taken by Syn-1 for the internalization of apoE-VLDL, we measured uptake of ^125^I-apoE-VLDL and ^125^I-α_2_M by GM00701/Syn-1-HA cells under conditions of K^+ ^depletion. Larkin, et al., have previously shown that K^+ ^depletion specifically inhibits clathrin-mediated internalization, but has little or no affect on non-clathrin mediated endocytosis [42]. As shown in Figure 7, K^+ ^depletion significantly inhibited ^125^I-α_2_M uptake by LRP as expected since LRP endocytosis is clathrin-mediated. However, ^125^I-apoE-VLDL internalization was unaffected by this treatment indicating that Syn-1 with bound apoE-VLDL is endocytosed through a non-clathrin mediated pathway.

**Figure 8:**
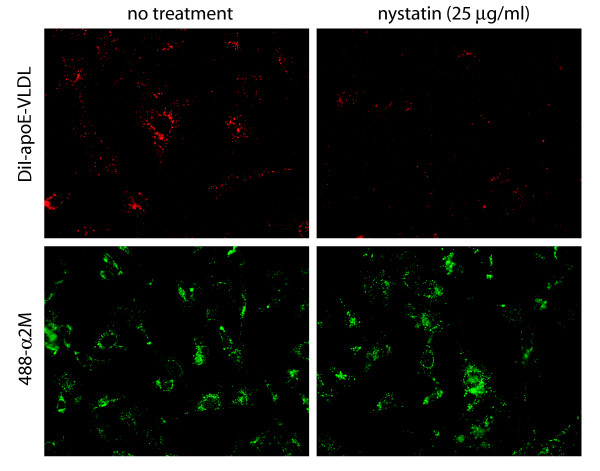
**Syn-1 internalizes DiI-apoE-VLDL through a lipid raft-mediated pathway**. GM00701/Syn-1-HA cells were incubated at 37°C in the absence or presence of nystatin (25 μg/ml) for 30 min prior to a 60 min incubation with DiI-apoE-VLDL (upper panels) or 488-α_2_M (lower panels) (5 μg/ml). Cells were fixed and visualized by fluorescence microscopy (488-α_2_M, FITC filter set; DiI-apoE-VLDL, rhodamine filter set). Magnification, 630X.

To determine if Syn-1 with bound apoE-VLDL internalizes through lipid raft domains, we examined the effect of nystatin on uptake of DiI-apoE-VLDL in GM00701/Syn-1-HA cells. Nystatin is a sterol-binding antibiotic that sequesters membrane-associated cholesterol thereby disrupting plasma membrane microdomains such as lipid rafts [43-45]. As shown in Figure 8, nystatin had no effect on clathrin-mediated endocytosis of a_2_M (lower panels). By contrast, little or no uptake of DiI-apoE-VLDL occurred in the presence of nystatin (upper panels) indicating that Syn-1 internalizes apoE-VLDL through a lipid raft microdomain.

## Discussion

Our previous study demonstrated that HSPG are able to bind and internalize apoE-VLDL with little or no contributions from LRP [16]. In the present study, we now explore if Syn-1 represents a class of HSPG that is able to provide a path for apoE-VLDL binding and internalization, and investigate its relationship with LRP in this function. Using GM00701 cells, a human fibroblast cell line that expresses abundant amounts of LRP and no detectable Syn-1, we show that these cells are unable to bind or internalize apoE-VLDL even with active LRP receptor function, which was confirmed by α_2_M binding and degradation. However, apoE-VLDL binding and internalization by these cells was rescued following the introduction of stable Syn-1 expression indicating that this HSPG is fully capable of mediating lipoprotein binding and uptake. We also show that apoE-VLDL internalization by the transfected cells is blocked by heparin co-incubation, but unaffected by a LRP ligand binding antagonist demonstrating that Syn-1 mediates uptake in an LRP-independent manner. To the best of our knowledge, this is the first example of a member of the syndecan family of HSPG serving as a clearance receptor for apoE-VLDL particles.

A previous study by Fuki, et al. [27], demonstrated that Syn-1 internalizes lipoprotein lipase-enriched LDL through a pathway that is distinct from clathrin coated pits and that internalization proceeds after clustering Syn-1 with a multivalent ligand. Since LRP apparently plays little role in the uptake of apoE-VLDL in GM00701 cells, we examined if internalization of apoE-VLDL by Syn-1 occurs through a clathrin-dependent or clathrin-independent pathway. We found that K^+ ^depletion significantly inhibited α_2_M uptake, which internalizes via LRP through a clathrin-mediated pathway, but uptake of apoE-VLDL was unaffected by this treatment. By contrast, treatment of cells with nystatin, which disrupts lipid raft function [43-45], prevented apoE-VLDL uptake by Syn-1 indicating that Syn-1-mediated clearance of apoE-VLDL proceeds through a non-clathrin pathway. This conclusion is also consistent with the fact that the cytoplasmic tail of Syn-1 does not contain the clathrin-mediated internalization signal sequence (NPXY). Additional evidence for non-clathrin-mediated internalization for Syn-1 was obtained through studies using a chimeric receptor consisting of the ectodomain of the Fc receptor Ia linked to the transmembrane and cytoplasmic domains of Syn-1 [28]. Upon clustering this chimera with IgG, it was found to redistribute into detergent insoluble lipid rafts. Pretreatment of cells with cholesterol depleting reagents prevented detergent insolubility of the Fc-Syn-1 chimera upon clustering and inhibited its internalization. From these data it appears that the transmembrane domain and cytoplasmic tail sequences of Syn-1 are sufficient for partitioning the receptor into lipid raft domains. Recently, it was shown that Syn-4 also redistributes into lipid rafts upon clustering [46], suggesting that this may be a conserved feature among all members of the syndecan family.

The mechanism of endocytosis by means of lipid raft domains remains a topic of ongoing study; however, information is becoming available as to the initial partitioning of proteins into lipid rafts [47,48]. Possible mechanisms to segregate plasma membrane proteins include hydrophobic modifications, such as lipidation, protein-protein interactions with lipid-raft resident proteins, or targeting signals that are encoded within the polypeptide domain of the membrane protein. Although Syn-1 has not been reported to undergo lipid modification or directly interact with lipid raft residents, such as caveolin, annexin, or flotillin, it may utilize intrinsic targeting information within its transmembrane domain or cytoplasmic tail in a similar fashion as the epidermal growth factor receptor [49]. Once receptors internalize with their bound ligand through non-clathrin mediated pathways, they can deliver their cargo to classic endocytic compartments, such as recycling endosomes [50]. As a result of this crosstalk between non-clathrin pathways and classical endocytic pathways, Syn-1 would be able to deliver apoE-VLDL to lysosomal acid lipases for release of fatty acids for cellular utilization [51]. Whether Syn-1 recycles to the cell surface after delivering its cargo to endosomes remains to be determined.

LRP was originally identified as a lipoprotein clearance receptor by virtue of its structural homology with the LDL receptor, its high level expression in liver and its function in hepatic remnant metabolism elegantly demonstrated using genetically modified mice [12,13]. However, the question remains as to why in certain cell types, HSPG, and as we show here, Syn-1, serves as the primary clearance receptor for apoE-VLDL and LRP plays little or no role in the uptake of this ligand. Answers to this question may come from more recent studies investigating alternative functions for LRP (reviewed in [52-54]). LRP is now known to carry out dual roles; serving as an endocytic receptor for many diverse, structurally different ligands and as a signaling receptor that is able to modulate activities of other cell surface proteins. Whether LRP functions as a cargo receptor or signaling receptor may be determined by the state of receptor phosphorylation, post-translational processing, or complement of cytosolic messengers/adaptors that might dictate the preference between these dual functions. Thus, studies on hepatocytes have demonstrated that LRP functions as a key element in lipoprotein handling *in vivo*, whereas in fibroblasts [55,56] or cells of neuronal lineage [57], LRP may be acting primarily as a co-receptor modulating signal transduction events that control cell behavior.

## Methods

### Materials

Human α_2_-macroglobulin (α_2_M), heparin, and heparinase were purchased from Sigma-Aldrich (St. Louis, MO). Na^125^I was purchased from Perkin-Elmer (Boston, MA). Pre-cast polyacrylamide electrophoresis (PAGE) gels and goat anti-rabbit horseradish peroxidase-conjugated secondary antibody were from BioRad (Hercules, CA). All oligonucleotide primers were synthesized by Integrated DNA Technologies (Coralville, IA). All restriction enzymes were from New England Biolabs (Ipswich, MA). RAP-GST fusion protein was purified as previously described [58]. Anti-LRP polyclonal antibody was raised against an 18 amino acid peptide from the cytoplasmic tail of human LRP [59]. Tissue culture plastics were purchased from Corning (Corning, NY). Buffers, salts, and detergents were obtained from either Sigma-Aldrich or Calbiochem (La Jolla, CA).

### Cell culture

GM00701 human fibroblasts (Coriell Institute, Camden, NJ) were obtained from a patient diagnosed with familial hypercholesterolemia. This cell line expresses < 1% of normal activity for the LDL receptor. GM00701 cells were cultured in Minimal Essential Media, Eagle modification with Earle's balanced salt solution (MEM-Eagle's, Life Technologies/Invitrogen, Carlsbad, CA). Media was supplemented with 2X concentration of essential and non-essential amino acids and vitamins, 15% (v/v) fetal bovine serum (Irvine Scientific, Santa Ana, CA), 100 μg/ml streptomycin sulfate, and 100 units/ml penicillin G. 3T3-L1 cells were obtained from American Type Culture Collection (Manassas, VA) and grown in Dulbecco's modified Eagle's medium (DMEM) (Invitrogen, Carlsbad, CA) supplemented with 10% (v/v) fetal calf serum (Irvine Scientific, Santa Ana, CA), 1 mM sodium pyruvate, 100 μg/ml streptomycin sulfate, and 100 units/ml penicillin. Cells were cultured at 37°C with 5% CO_2 _for GM00701 cells and 10% CO_2 _for preadipocyte 3T3-L1 cells. Cells were passaged twice weekly.

### Immunoblotting

Cell lysates were prepared with 20 mM Tris pH 7.4, 150 mM NaCl (TBS) containing either 1% (v/v) Triton-X100 or 8 M urea. Solubilized proteins were mixed with SDS-PAGE sample buffer supplemented with 2% (v/v) β-mercaptoethanol, heated to 95°C, separated by SDS-PAGE and transferred to Immobilon-P (Millipore, Billerica, MA) using a wet tank transfer system (BioRad, Hercules, CA). Membranes were blocked with TBS, 0.1% (v/v) Tween-20, 5% (w/v) non-fat dry milk for 20 minutes at 23°C and incubated with the indicated primary antibody for 2 h at 23°C. Membranes were washed three times (10 min each) with TBS, 0.1% (v/v) Tween-20, and bound antibodies were detected with species-specific HRP-conjugated secondary antibodies (1:3000, BioRad) followed by chemiluminescence detection according to the manufacturer's instructions (Pierce, Rockford, IL). Images were captured using a Syngene GeneGnome system equipped with a Peltier-cooled 16-bit CCD camera and saturation detection. Densitometry of scanned images was performed using Scion Image software version 4.0.2.

### Activation of α_2_-macroglobulin and labeling procedures

α_2_M was activated for receptor binding by incubating with an equal volume of 0.4 M methylamine in 0.1 M Tris-HCl, pH 8, for 2 h at room temperature. Unbound methylamine was then removed by passage over a desalting column (PD-10, Amersham Biosciences). For radioiodinations, both apoE-VLDL and α_2_M were labeled with ^125^I (Perkin-Elmer) using Iodo-Beads (Pierce) as previously described [60]. Specific activities were routinely between 3000 and 5000 cpm/ng protein. Using the Bligh-Dyer lipid extraction procedure, we determined that approximately 10% of ^125^I-label was incorporated into lipid of the VLDL particle while the remaining 90% of label was found coupled to protein. α_2_M was labeled for fluorescence detection using Alexa Fluor^® ^488 Protein labeling kit (Molecular Probes, Eugene, OR).

### Cell surface 4°C binding assay

Cells were grown on tissue culture plates precoated with 1% gelatin and used when confluent. Cells were rinsed twice with 20 mM Hepes, pH 7.4, 150 mM NaCl, 2 mM CaCl_2 _(buffer A), followed by incubation for 3 h with ^125^I-apoE-VLDL (2 μg/ml) or ^125^I-α_2_M (1 μg/ml) at 4°C in the absence or presence of the indicated unlabeled competitors. ^125^I-ligands were diluted into buffer A containing 1% BSA and chilled to 4°C before adding to cells. Unbound ^125^I-ligand was removed by rinsing cells three times with cold buffer A after which cells with bound ligand were solubilized with 0.1 N NaOH. Solubilized proteins were added to EcoLume (ICN Biomedicals, Costa Mesa, CA) and subjected to scintillation counting (73% efficiency for ^125^Iodine). Results were normalized to total cellular protein (BCA Protein Assay, Pierce, Rockford, IL). Specificity was determined as the difference between total binding (without competition) and non-specific binding (non-competable) [61]. The actual amount of ligand bound to cells was calculated as cpm ÷ the specific activity of ^125^I-labeled ligand. All data points represent averages of replicate points with standard errors of < 5%.

### 37°C ligand degradation assay

Cells were incubated at 37°C/5% CO_2 _with ^125^I-α_2_M (1 μg/ml) diluted into MEM-Eagle's containing 1% BSA in the absence or presence of unlabeled α_2_M (10 μg/ml). At the indicated times, media was removed and processed for trichloroacetic acid (TCA) precipitation [62]. TCA soluble material was added to EcoLume and subjected to scintillation counting. Degradation was calculated as TCA-soluble cpm ÷ specific activity of the ^125^I-α_2_M.

### pMH/Syn-1-HA construction and GM00701 cell transfections

The cDNA for full length human Syn-1 was obtained from the I.M.A.G.E. Consortium (clone no. 3347793 in pOTB7 vector, accession no. BE272506) and amplified by PCR using the following conditions: 30 cycles, 94°C for 1 min, 60°C for 1 min, 72°C for 3 min; 1 cycle, 72°C for 7 min. Primer sequences used: upstream, 5'-CCGGGCAGCATGGGGCGCGCG-3'; downstream, 5'-TTGGCATAGAATTCCTCCTGTTTGG-3'. The cDNA was cloned into pMH vector (Roche, Indianapolis, IN) using restriction sites HindIII and Not1, and placed in frame with a hemagglutinin (HA) peptide-encoding sequence at its 3' end. pMH/Syn-1-HA vector was introduced into DH5α E. coli, amplified by overnight growth, and purified for mammalian transfections using an endotoxin-free plasmid purification kit (Qiagen, Valencia, CA). For transfections, GM00701 cells were plated onto 10 cm tissue culture dishes and incubated with 8 μg vector/dish together with Lipofectamine Plus according to the manufacturer's instructions (Invitrogen, Carlsbad, CA). Forty eight hours post-transfection, cell growth media was replaced with fresh media containing 1 mg/ml neomycin for selection of stably transfected cells.

### Anti-Syn-1 polyclonal antibody

The cDNA for full length human Syn-1 was amplified by PCR using the conditions described above and cloned into pET28 vector (EMD Biosciences, San Diego, CA) using restriction sites HindIII and XhoI. The cDNA was placed in frame with a hexa-histidine encoding sequence at its 3' end (Syn-1-His_6_). Primer sequences used: upstream, 5'-GCAAGCTTCAAATTGTGGCTACTAATTTGCCC-3'; downstream, 5'-CGCTCGAGGGCATAGAATTCCTCCTGTTTGG-3'. Syn-1-His_6 _was purified by nickel-affinity chromatography and purity (> 95%) was confirmed by Coomassie staining following SDS-PAGE. Protein identity was also confirmed by mass spectrometry analysis (University of New Mexico Proteomics Core Facility). Purified Syn-1-His_6 _(500 μg) was thoroughly mixed with an equal volume of Freund's Complete adjuvant and injected subcutaneously into New Zealand White rabbits. Rabbits were boosted with 100 μg Syn-1-His_6 _mixed with Freund's Incomplete adjuvant at three week intervals.

### Preparation of Di-I-labeled apoE-VLDL

New Zealand White rabbits were placed on a high-fat chow diet (10% peanut oil/1% cholesterol) for a minimum of 4 d, blood was drawn into 1 mM EDTA and centrifuged at 2000 × g, 15 min to remove cells. Chylomicrons were floated by centrifuging plasma at 100,000 × g for 10 min and removed by pipetting. Plasma was then mixed with OptiPrep™ (12% iodixanol final concentration, Oslo, Norway) and centrifuged at 350,000 × g for 20 h (SW55Ti rotor) with slow acceleration and deceleration. VLDL particles (density of 1.006 g/ml) were removed from the top of the gradient by pipetting and analyzed by SDS-PAGE and Coomassie R staining to confirm the presence of apoB100 (Mr, 512 kDa) and apoE (Mr, 34 kDa). Animal protocol (#0350) was approved by the University of New Mexico, Health Sciences Center Laboratory Animal Care and Use Committee. For DiI (3,3'-dioctadecylindocarbocyanine, Molecular Probes, Invitrogen) labeling, a working stock of 3 mg/ml was made in dimethylsulfoxide and 0.15 mg was slowly added to 1.67 mg apoE-VLDL (in 1.9 ml) with vortexing to rapidly mix. The mixture was then wrapped in foil and incubated for 8 h at 37°C. Unincorporated DiI was removed from DiI-labeled apoE-VLDL by OptiPrep™ gradient centrifugation as described above.

### DiI-labeled apoE-VLDL uptake assay

GM00701 or GM00701/Syn-1-HA cells were plated on glass coverslips, rinsed twice with MEM-Eagle's and incubated with DiI-apoE-VLDL (4 μg/ml) diluted into MEM-Eagle's containing 1% bovine serum albumin in the absence or presence of heparin (200 μg/ml) or RAP-GST (50 μg/ml). After 1 h at 37°C, cells were rinsed with phosphate buffered saline, fixed with 3% (w/v) paraformaldehyde for 20 min, and mounted using Vectashield (Vector Labs, Burlingame, CA). Cells were observed with a Zeiss Axioskop microscope equipped for epifluorescence. Images were capture with a Hamamatsu digital/video camera and AxioVision software.

### K^+ ^depletion studies

K^+ ^depletion studies were carried out according to Larkin, et al. [42]. Briefly, GM00701/Syn-1-HA cells were rinsed with K^+^-free buffer (50 mM Hepes, pH 7.4, 100 mM NaCl) and subjected to hypotonic shock by incubation at 37°C for 5 min with a 1:1 ratio of MEM-Eagle's media:dH_2_O. Hypotonic shock buffer was then replaced with K^+^-free buffer for 10 min at 37°C. Control, non-treated cells were incubated in parallel with serum-free, MEM-Eagle's media. Cells were then incubated at 37°C with either ^125^I-apoE-VLDL or ^125^I-α_2_M diluted into K^+^-free buffer (treated cells) or serum-free, MEM-Eagle's media (control cells). At the indicated times, cells were chilled on ice and rinsed (3X) with ice cold 20 mM Hepes, pH 7.4, 150 mM NaCl. Bound ligand that had not undergone internalization was dissociated by a 5 min incubation with 20 mM NaOAc, pH 3, 150 mM NaCl. Internalized ^125^I-ligand was then extracted by solubilizing cells with 0.1 N NaOH, added to EcoLume and quantitated by scintillation counting. In all cases, specificity of ligand internalization was determined by a parallel co-incubation with > 10-fold molar excess of unlabeled ligand.

## Abbreviations

ApoE = apolipoprotein E

HSPG = heparan sulfate proteoglycan

VLDL = very low density lipoprotein

LRP = low density lipoprotein receptor-related protein

Syn-1 = syndecan-1

RAP = receptor associated protein

LPL = lipoprotein lipase

DiI = 1,1'-dictadecyl-3,3,3',3'-tetramethylindocarbocyanine perchlorate

## Competing interests

The author(s) declare that they have no competing interests.

## Authors' contributions

LCW carried out the majority of studies. AMG contributed to ligand uptake studies and manuscript preparation. RAO provided the original conceptual framework for the study, carried out pilot experiments, organized experimental design and finalized the manuscript for submission. All authors read and approved the final version.
